# Assessing the User Experience of the EU Mobile App for Cancer Prevention: Mixed Methods Study

**DOI:** 10.2196/73844

**Published:** 2025-09-19

**Authors:** Furqan Ahmed, Silvana Romero Saletti, Erica D’Souza, Carolina Espina, David Ritchie, Ana Molina Barceló, Marina Pinto Carbó, Paula Romeo Cervera, Teresa Seum, Hermann Brenner, Stephan Van den Broucke, Maria Krini, Cristiana Fonseca, Patricia Pinto, Diana Krivic, Helena Ros Comesana, Wendy Yared, Hajo Zeeb, Tilman Brand

**Affiliations:** 1 Department of Prevention and Evaluation, Leibniz Institute for Prevention Research and Epidemiology, Achterstraße 30, Bremen, 28359, Germany, 49 42121856913; 2 Psychological Sciences Research Institute, Université Catholique de Louvain, Louvain-la-Neuve, Belgium; 3 International Agency for Research on Cancer, World Health Organization, Lyon, France; 4 Cancer and Public Health Research Unit, The Foundation for the Promotion of Health and Biomedical Research of Valencia Region, Valencia, Spain; 5 Division of Clinical Epidemiology and Aging Research, German Cancer Research Center, Heidelberg, Germany; 6 Pagkyprios Syndesmos Karkinopathon Kai Filon, Paralimni, Cyprus; 7 Liga Portuguesa Contra o Cancro, Lisbon, Portugal; 8 Association of Slovenian Cancer Societies, Ljubljana, Slovenia; 9 Association of European Cancer Leagues, Brussels, Belgium; 10 Health Sciences Bremen, University of Bremen, Bremen, Germany

**Keywords:** cancer prevention, mobile application, usability testing, digital health literacy, European Code Against Cancer

## Abstract

**Background:**

In 2022, nearly 20 million new cancer cases and 9.7 million deaths occurred globally. Europe, comprising under 10% of the world’s population, accounted for over 22% of cases and 20% of deaths, reflecting an aging population, lifestyle risk factors, and extensive screening. With 40% of cancers preventable through modifiable risk factor interventions, effective prevention is essential. The European Code Against Cancer provides evidence-based guidelines that drive health initiatives across Europe. Supported by Europe’s Beating Cancer Plan and the EU4Health program, the EU Mobile App for Cancer Prevention was developed to disseminate these recommendations. However, its effectiveness depends on usability across populations with varying digital and health literacy; this study evaluates the app’s usability among diverse European populations.

**Objective:**

This study aimed to identify enablers, barriers, and user requirements for the use and maintenance of the English version of the EU Mobile App for Cancer Prevention, focusing on how usability varied across individuals with different levels of digital health literacy and diverse sociodemographic backgrounds. In addition, user feedback on mock wireframes—visual representations of the app’s interface and functionality—was gathered to evaluate usability and ease of use, providing insights for tailoring the app design to a broader population.

**Methods:**

We conducted a mixed methods study in 7 European countries with 76 adults aged 19‐84 years recruited via purposive quota sampling. Participants completed quantitative usability testing using mock wireframes to perform 10 predefined tasks simulating core app functionalities (eg, profile setup and health goal tracking). We recorded task completion time, success rates, self-reported confidence, and perceived difficulty. Digital health literacy was assessed using the eHealth Literacy Scale (eHEALS) scale. Qualitative data were collected through focus group discussions guided by a semistructured interview guide, and transcripts were analyzed via thematic content analysis. Statistical analyses included descriptive statistics and 1-way ANOVA to explore group differences.

**Results:**

Overall task completion rates ranged from 75% to 98%, with a median of 86%, indicating general usability. However, usability varied by age, education, and digital health literacy: younger participants and those with higher education and literacy levels reported greater confidence and lower difficulty, whereas older adults and lower-literacy users experienced more challenges. Qualitative analysis identified key themes affecting usability: the need for accessibility (multilingual support and simple language), user-centric design (age-friendly interfaces and intuitive navigation), ethical concerns (data privacy and security), and motivational features (gamification and personalized health goals).

**Conclusion:**

The app is generally usable across diverse populations but requires streamlined interfaces and design adaptations to accommodate varying digital health literacy. Ensuring robust data privacy practices is essential for fostering user trust, and integrating motivational elements may enhance sustained engagement. Future work will involve piloting the finalized app to evaluate its real-world uptake and impact on cancer prevention behaviors.

## Introduction

According to the most recent Global Cancer Observatory database (GLOBOCAN) 2022 estimates, nearly 20 million new cancer cases (including nonmelanoma skin cancers) and approximately 9.7 million cancer deaths were recorded worldwide in 2022 [1]. Although Europe comprises less than 10% of the global population, it accounts for over 22% of new cancer cases and about 20% of cancer deaths driven largely by its aging demographic, lifestyle risk factors, and extensive screening protocols [1]. Notably, lung cancer remains the leading cause of cancer morbidity and mortality, responsible for roughly 12.4% of new cases and 18.7% of cancer deaths globally. With demographic projections indicating that annual new cancer cases could surge to 35 million by 2050—a 77% increase from 2022—there is an urgent need for comprehensive prevention strategies, equitable health policies, and rigorous research to reduce future cancer burdens globally [1].

An estimated 40% of cancer cases are preventable through interventions targeting modifiable risk factors, reinforcing the importance of strong preventive measures [2]. The European Code Against Cancer (ECAC), an initiative launched by the European Commission over 3 decades ago and coordinated by the International Agency for Research on Cancer (IARC), provides a framework of evidence-based recommendations to reduce cancer risk [3]. The ECAC fourth edition, introduced in 2014, outlines 12 actionable guidelines that range from limiting exposure to recognized carcinogens to embracing health-promoting behaviors and participating in organized screening [3]. These recommendations guide health promotion initiatives across Europe, supported by civil society organizations, national and regional cancer leagues, and public health authorities [4].

In 2021, the European Commission launched Europe’s Beating Cancer Plan, emphasizing the critical need to support cancer-related health literacy [5]. Funding from the EU4Health program supports the implementation of ECAC guidelines, particularly among vulnerable and underserved communities [5]. As part of these efforts, the Commission financed the EU Mobile App for Cancer Prevention to broaden ECAC’s reach [6]. In recent years, mobile health apps have emerged as promising tools for cancer prevention, offering interactive platforms to deliver lifestyle interventions and educational content. For example, a systematic review reported that such mobile app interventions are being used to support cancer prevention and early detection in diverse global contexts, including low- and middle-income countries [7]. Within Europe, researchers have also tailored apps for specific populations; a notable case is an adolescent-focused app based on the ECAC recommendations, which demonstrated high user engagement through gamified modules and provided valuable insights for the development of the EU Mobile App [8].

Health literacy—the ability to access, understand, and apply health information to make informed decisions—has expanded in scope to include digital health literacy (DHL), which encompasses the skills needed to navigate, evaluate, and act on health information through digital platforms [9,10]. Together, these competencies are critical determinants of cancer prevention outcomes, shaping an individual’s capacity to adopt preventive behaviors, interpret risk factors, and engage with screening programs. Digital tools, such as mobile apps, offer opportunities to democratize access to evidence-based health information across diverse populations. However, without purposeful design, these tools risk perpetuating or deepening existing inequities, as disparities in health literacy and DHL intersect with socioeconomic, age-related, and technological barriers. Within the EU, approximately 10% of the population struggles with inadequate health literacy [9], while far more face challenges in DHL, particularly older adults, rural communities, and marginalized groups. Limited health literacy is associated with delayed cancer screenings, poor adherence to preventive guidelines, and higher mortality rates [10]. Low DHL can limit access to digital resources, spread misinformation, and reduce trust in online health content [11,12]. Improving both health literacy and DHL is thus crucial for equitable cancer prevention.

In response to these challenges, the Boosting the Usability of the EU Mobile App for Cancer Prevention (BUMPER) project was initiated in November 2022 [6]. In this paper, we describe the pilot usability testing of the EU Mobile App for Cancer Prevention. The study focused on app usability among individuals with varying levels of DHL, seeking to enhance the app’s appropriateness in supporting cancer prevention across heterogeneous communities. Participants were asked to complete 10 tasks using mock wireframes of the app, followed by focus group discussions (FGDs) that provided extensive insights into enablers, barriers, and user requirements.

## Methods

### Aim of the Study

This study aimed to identify enablers, barriers, and user requirements associated with the use and maintenance of the EU Mobile App for Cancer Prevention (English version). Specifically, the investigation explored how app usability varied across individuals with different levels of DHL and diverse sociodemographic backgrounds. In addition, user feedback on mock wireframes (visual representations of the app’s interface and functionality) was gathered to evaluate usability and ease of use.

### Study Design

A mixed‐methods design was implemented, combining quantitative pilot testing of wireframes with qualitative FGDs to assess app usability [13]. This approach enabled the collection of both measurable usability metrics and in‐depth qualitative insights into user perspectives [14,15]. We adhered to the STAndards for Reporting of Evaluation studies in Health Informatics (STARE-HI) reporting guidelines for health informatics studies; the completed STARE-HI checklist is provided in Multimedia Appendix 1.

### Study Setting

The study was conducted in 7 European countries: Cyprus, Finland, Germany, Hungary, Portugal, Slovenia, and Spain. Data collection included in-person sessions with local Cancer Leagues in Cyprus, Finland, Hungary, Portugal, Slovenia, and Spain. In Germany, the Leibniz Living Lab Bremen (part of the Leibniz Institute for Prevention Research and Epidemiology, BIPS) supported the pilot study [16,17]. In Cyprus, limited space ruled out group meetings, so data was collected through individual interviews. A total of 2 FGDs were conducted per country, with individual interviews and pilot testing in Cyprus. Study sites and countries were chosen based on the presence of BUMPER consortium partners; accordingly, in Germany, all data were collected at the Living Lab, rather than a cancer league, since only project partners conducted evaluations.

### Participant Eligibility Criteria

Participants were included if they (1) were consenting adults (aged 18 y and older) residing in one of the participating countries, (2) owned a smartphone or tablet with internet access (required for engaging with the wireframes), and (3) could communicate in English or the local language.

### Sampling and Recruitment

A total of 2 FGDs, each comprising 8‐12 participants, were planned per country. A quota sampling strategy was used to ensure representation across sociodemographic backgrounds, education levels, sex, and age groups [18]. Participants were stratified into three predefined categories: (1) age (18‐40, 41‐60, and ≥61 y), (2) gender, and (3) educational attainment (low: ≤12 y of schooling and high: university degree or higher). Recruitment was facilitated through collaborations with local organizations, including national cancer leagues, to ensure diverse participation across all 7 countries. Further details of the sampling framework are provided in Multimedia Appendix 1.

### Data Collection

Moderators received training on both FGD facilitation and wireframe usability testing in collaboration with the app’s technical developer [13,19]. Detailed training manuals provided questionnaires, wireframe tasks, and reporting templates. A total of 3 training sessions were conducted in October 2023: 2 covering FGDs (led by BIPS) and 1 led by the app developer on wireframe implementation and evaluation.

Data were collected from October 2023 to November 2023. Before usability testing, participants completed a sociodemographic questionnaire and the eHealth Literacy Scale (eHEALS) to measure DHL [20]. They then completed 10 predefined tasks using an English-language mock-up of the app (see Table 1). These tasks addressed essential functionalities such as user profile setup, health goal tracking, and accessing cancer prevention resources. Participants scanned QR codes and followed on-screen instructions for each task. Afterward, they rated confidence and perceived difficulty on Likert scales, while trained observers noted usability issues or challenges [19]. Usability metrics included task completion time and success or failure status, as recorded by moderators.

Wireframes were fully interactive app prototypes on participants’ smartphones, mirroring the look, feel, and navigation of the final app, and thus, task completion times provide a valid indicator of actual interaction difficulty and cognitive load. Reporting time on task alongside confidence and difficulty ratings enriched our understanding of usability by quantifying how long users take to locate features under realistic conditions. The EU Mobile App for Cancer Prevention was organized into 4 main tabs, “Dashboard,” “Goals,” “Learning,” and “Profile,” each accessed via a bottom navigation bar. The “Dashboard” greeted users by name and visualized real-time progress on personalized prevention goals through simple charts and daily 1-tap logs (eg, tobacco use and physical activity). In “Goals,” users browsed suggested targets, monitored active objectives, and reviewed completed milestones via clear icons and progress bars. The “Learning” tab featured a “Discover” feed of evidence-based topics (eg, smoking cessation and healthy weight) directly linked to the European Code Against Cancer’s “12 Ways” guidelines. Finally, “Profile” allowed users to input age, sex, and health data to tailor content delivery and reminders.

**Table 1.** Task details and success criteria.

Task	Description	Success criteria (Expected outcome)	Completion status	Additional details
Task 1: Profile set up	Download the app and set up your personal profile to receive personalized recommendations.	Land on the profile setup screen.	Pass or fail	Time tracked; trainer notes available.
Task 2: Set initial goal	Set a goal for smoking reduction based on app-suggested goals.	Land on the goal-setting screen.	Pass or fail	Time tracked; trainer notes available.
Task 3: Goal progress	Find an active goal and a successfully completed goal.	Identify “Smoking reduction” or “Weight loss” as goals.	Pass or fail	Time tracked; trainer notes available.
Task 4: Daily tracking	Track your daily progress toward active goals.	Locate the recommendation “Avoid being outside.”	Pass or fail	Time tracked; trainer notes available.
Task 5: UV Index	Check if conditions are ideal for outdoor activities.	Land on the UV Index screen and click “SAVE.”	Pass or fail	Time tracked; trainer notes available.
Task 6: Goal management	Find tips on achieving the “Smoking reduction” goal.	Locate the “Practical tips” section.	Pass or fail	Time tracked; trainer notes available.
Task 7: Challenge a friend	Challenge a friend to the “Smoking reduction” goal.	Land on the challenge screen and click “SEND.”	Pass or fail	Time tracked; trainer notes available.
Task 8: Reminders	Find upcoming reminders and create a new one for breast screening.	Land on the reminder screen and set a new reminder.	Pass or fail	Time tracked; trainer notes available.
Task 9: Learning	Find articles about smoking with the World Health Organization as the source.	Select “Smoking” and “World Health Organization” under the categories.	Pass or fail	Time tracked; trainer notes available.
Task 10: Awards	Find the criteria or steps needed to earn the “Smoke Warrior” award.	Identify “Be smoke-free for 12 months” as the criteria.	Pass or fail	Time tracked; trainer notes available.

Following usability testing, participants joined FGDs to explore their experiences, challenges, and perceptions of the app. A semistructured interview guide, based on the Leibniz ScienceCampus Digital Public Health framework, ensured methodological consistency throughout the sessions [17,21]. All discussions were audio-recorded to ensure comprehensive data capture [22]. The interview guide is provided in Multimedia Appendix 1.

### Data Analysis: Quantitative Analysis

Descriptive statistics summarized categorical variables (country, biological sex, education level, disability status, and task completion) and continuous variables (age, eHEALS scores, and task completion times), along with Likert scale responses. The eHEALS comprises 8 items scored on a 5-point Likert scale (1=strongly disagree and 5=strongly agree), total scores from 8 to 40 (higher scores represent higher self-rated DHL). Key variables were categorized as follows: education level (low: no formal education, primary, lower, or upper secondary and high: trade, technical or vocational, bachelor’s degree, master’s degree, or doctorate); median-split DHL scores (low vs high DHL), using an eHEALS median of 30.5 as the cutoff; and 4 age groups (young adults: 18‐40 y, middle-aged adults: 41‐60 y, and older adults: ≥61 y). We dichotomized eHEALS scores at the sample median (30.5) because no validated threshold exists for “high” versus “low” digital health literacy, yielding 2 equal‐sized groups for robust 1‐way ANOVA comparisons. Furthermore, 1-way ANOVAs assessed the association of these factors on continuous outcomes (eg, task completion time, confidence, perceived difficulty, and likelihood of future use) [20]. A series of 1-way ANOVAs using ordinary least squares determined statistical significance at *P*<.05. Group means were computed for interpretation. To validate the ANOVA findings, assumptions were checked: the Shapiro-Wilk and Q-Q plots for normality, the Levene test for homogeneity of variance, and the Cook distance (<1) for outlier detection [23]. Statistical analyses were performed using Python (version 3.11.8; Python Software Foundation) with *Pandas*, *NumPy*, *SciPy*, *Statsmodels*, and *Seaborn* packages [24,25].

### Data Analysis: Qualitative Analysis

All FGDs and interviews were audio-recorded and later transcribed verbatim by professional transcription services. Artificial intelligence (AI)–assisted software supported translations into English, with local teams verifying translation accuracy. Thematic content analysis followed Bengtsson’s framework [26], encompassing decontextualization, recontextualization, categorization, and compilation. Transcripts were imported into ATLAS.ti software (version 9.22.0–2025-08-26; ATLAS.ti Scientific Software Development GmbH), which used AI-driven natural language processing to expedite coding [26-28]. The final coding scheme emerged from a combination of open coding and research question–driven coding, with 2 reviewers reconciling any discrepancies to ensure reliability. Multiple researchers subsequently evaluated the findings to increase validity and capture nuanced interpretations [26-28].

### Ethical Considerations

The BUMPER pilot study obtained ethical clearance from the Ethics Commission at the University of Bremen (Application 2023‐10). All participants provided informed consent before enrolling, with assurance of confidentiality and anonymity in published findings. No participants are identifiable in any images or results presented, and participants received no monetary compensation beyond reimbursement of travel expenses (only by some countries).

## Results

### Demographic Characteristics

A total of 76 participants from 7 European countries (Germany: n=13, 17%; Finland, Hungary, and Spain: n=12, 16% each; Portugal and Slovenia: n=10, 13% each; and Cyprus: n=7, 9%) were enrolled, with a near-balanced gender distribution (women: n=40, 53% and men: n=36, 47%). Age groups spanned 18‐84 years (mean 46, SD 17.3 y), though representation skewed toward younger adults (18‐40 y: n=30, 39%) compared to middle-aged (41‐60 y: n=25, 33%) and older adults (≥61 y: n=21, 28%). Education levels reflected stratification into “low” (≤12 years: n=33, 43% [primary: n=1, 1%; lower secondary: n=16, 21%; and upper secondary: n=15, 20%]) and “high” (postsecondary: n=43, 56% [vocational: n=7, 9%; bachelor’s: n=18, 24%; master’s: n=17, 22%; and doctorate: n=1, 1%])**,** with minimal representation of no formal education (n=1, 1%). Disabilities were reported by n=15 (20%) participants, primarily chronic illnesses (n=8, 11%), while n=61 (80%) reported no disability. Despite logistical constraints necessitating convenience sampling, the cohort broadly reflected the intended diversity in age, gender, and education. Table 2 shows these demographic characteristics.

**Table 2.** Participant demographics by country, gender, education level, and disability status.

Characteristic		Frequency, n (%)^a^	
Age group category			
Young adults (18‐40 y)		30 (39)	
Middle-aged adults (41‐60 y)		25 (33)	
Older adults (≥61 y)		21 (28)	
Country			
Germany		13 (17)	
Finland		12 (16)	
Hungary		12 (16)	
Spain		12 (16)	
Portugal		10 (13)	
Slovenia		10 (13)	
Cyprus		7 (9)	
Gender			
Women		40 (53)	
Men		36 (47)	
Other		0 (0)	
Education level			
Doctorate degree		1 (1)	
Master’s degree		17 (22)	
Bachelor’s degree		18 (24)	
Trade, technical, or vocational training		7 (9)	
Upper secondary education		15 (20)	
Lower secondary education		16 (21)	
Primary education		1 (1)	
No formal education		1 (1)	
Disability status			
No disability		61 (80)	
Chronic illness or autoimmune disorder (eg, diabetes or multiple sclerosis)		8 (11)	
Visual impairment (eg, low vision)		3 (4)	
Hearing impairment (eg, deafness or hard of hearing)		1 (1)	
Physical disability (eg, mobility impairment or amputation)		1 (1)	
Physical disability, mental health conditions, chronic illness, or autoimmune disease		1 (1)	
Mental health condition (eg, anxiety, depression, or bipolar disorder)		1 (1)	

a All percentages are based on a total of 76 observations per category. Rounding may cause totals not to sum exactly to 100%.

### Digital Health Literacy

**Figure 1.**
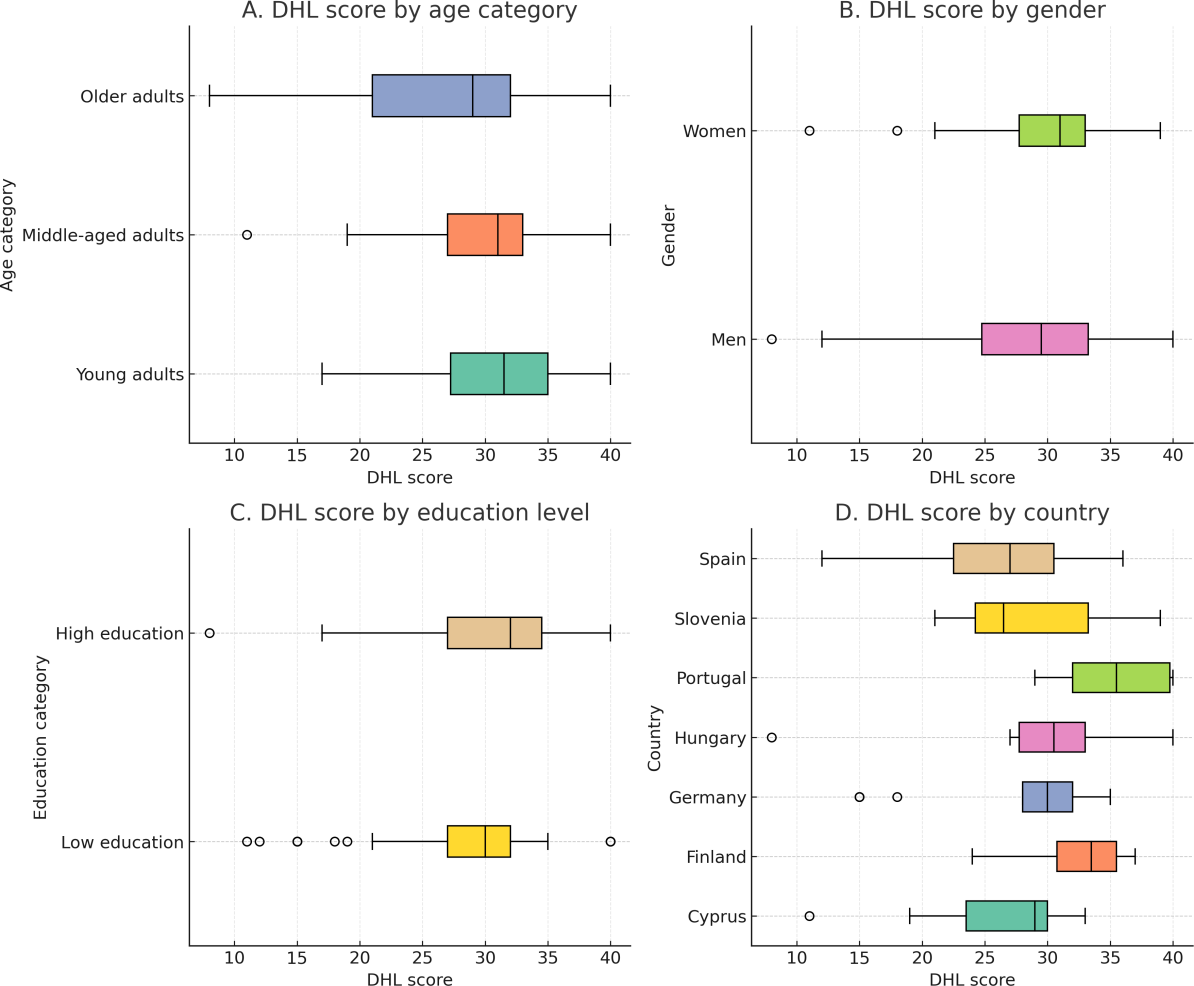
This figure presents the distribution of digital health literacy (DHL) scores across different sociodemographic categories, represented in 4 boxplots: (A) illustrates the DHL scores for 4 distinct age categories: young adults (18‐40 years), middle-aged adults (41‐60 years), and older adults (≥61 years); (B) shows the scores by sex, comparing women and men; (C) displays DHL scores based on education level education level (low: no formal education, primary, lower, or upper secondary; high: trade, technical or vocational, bachelor’s degree, master’s degree, or doctorate); and (D) compares the DHL scores by country, reflecting the various countries represented in the dataset.

Participants’ DHL was assessed using the eHEALS [20]. As depicted in Figure 1, DHL scores varied across sociodemographic groups. The overall DHL score averaged 29.4 (SD 7.0), with a median split at 30.5 categorizing participants into low and high DHL groups. Overall, the DHL scores show differences by age, sex, education, and country (see Figure 1). The analysis shows that younger adults (n=30) have a median DHL score of 31.5 (range 17-40; mean 31.0, SD 6.1), whereas older adults (n=21) have a lower median of 29.0 (range 8-40; mean 26.1, SD 8.1). Women exhibit a slightly higher median of 31.0 (n=40; range 11-39; mean 30.2, SD 5.7) compared to 29.5 median for men (n=36; range 8-40; mean 28.4, SD 8.1). Participants with higher education (n=43) demonstrate a median of 32.0 (range 21-40; mean 30.6, SD 6.5) relative to 30.0 for those with lower education (n=33; range 11-40; mean 27.8, SD 7.3). Portugal (n=10) among countries had the highest median DHL score at 35.5 (range 29-40; mean 35.3, SD 4.5), whereas Spain (n=12; median 27.0; range 15-35; mean 27.7, SD 6.9) and Cyprus (n=7; median 29.0; range 11-33; mean 25.7, SD 7.9) ranked lower.

### Time Spent on Tasks, Completion Status, Confidence, Difficulty Ratings, and Likelihood of Use

**Figure 2.**
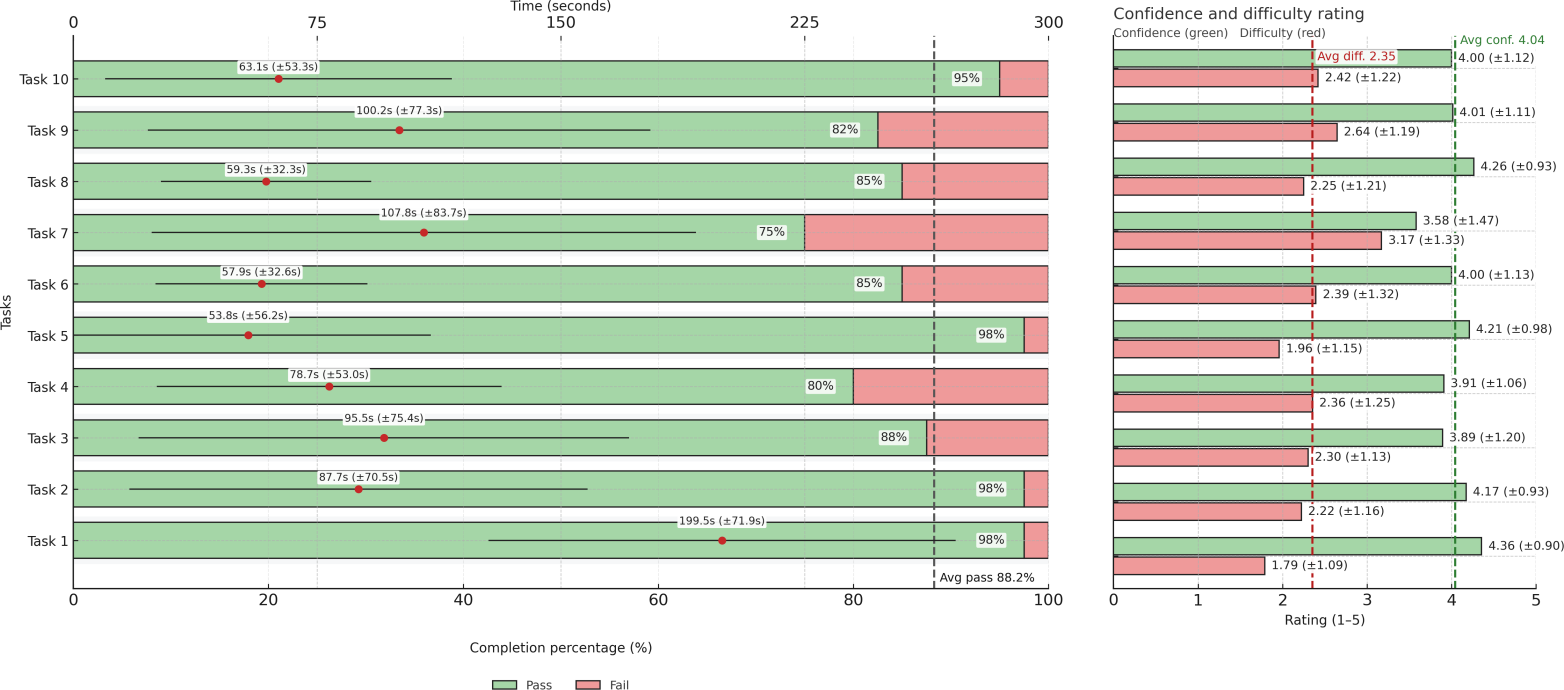
This figure presents task completion percentages, completion times, confidence, and difficulty ratings for each task, together with the overall averages. Each horizontal bar shows the proportion of participants who passed (green) or failed (red). Red dots on the bars represent the mean time to complete each task, with black whiskers indicating the SD of completion time. To the right, confidence and difficulty ratings are displayed with their respective SDs. Confidence ratings are shown in green bars (higher values=greater confidence), while difficulty ratings are shown in red bars (higher values=greater perceived difficulty). Dashed vertical lines mark the overall averages for confidence and difficulty, with the corresponding average values annotated. The average pass rate across all tasks is also indicated by a dashed line in the completion panel. Avg conf: average confidence; Avg diff: average difficulty.

Mean task completion times ranged from 54 seconds (Task 5) to 200 seconds (Task 1). Task 7 took an average of 108 seconds, with a maximum of 420 seconds, suggesting variability in perceived complexity. Task pass rates ranged from 75% (Task 7) to 98% (Tasks 1, 2, and 5), with a median completion rate of 86%. Mean confidence ratings (on a 5-point Likert scale) were generally high (3.6‐4.4), while mean difficulty ratings spanned 1.8‐3.2. Task 7 had both the lowest pass rate and the highest difficulty. Participants rated their likelihood of future app use at a moderate level (mean 3.5, SD 1.0). Figure 2 shows task completion percentages, time taken, confidence, and difficulty ratings for each task, along with the overall averages.

### ANOVA

A 1-way ANOVA examined the relationships between age, education level, or DHL and task-related outcomes (confidence and perceived difficulty). Assumptions of normality (Shapiro-Wilk test and Q-Q plots), homogeneity of variances (the Levene test), and absence of influential outliers (the Cook distance <1) were confirmed for all analyses unless otherwise specified.

#### Age Category

##### Average Time

Age category was significantly associated with average time spent per task (*F*_2,72_=3.6; *P*=.04). Young adults spent significantly less time (mean 75.4, SD 34.6) compared to middle-aged adults (mean 97.0, SD 40.7) and older adults (mean 111.2, SD 21.7).

##### Confidence in Tasks

Age category was significantly associated with confidence (*F*_2,72_=10.2; *P*<.001). Young adults (mean 4.4, SD 0.5) reported significantly higher confidence compared to middle-aged adults (mean 4.0, SD 0.6) and older adults (mean 3.6, SD 0.7).

##### Perceived Difficulty

Age category was significantly associated with perceived difficulty (*F*_2,72_=3.7; *P*=.03). Young adults reported significantly less difficulty (mean 2.1, SD 0.8) compared to middle-aged adults (mean 2.4, SD 0.7) and older adults (mean 2.7, SD 0.6).

### Education Level

Education was categorized as low (≤12 y of schooling: primary, lower, or upper secondary) or high (trade, technical, or vocational training or postsecondary degree). Participants with high education reported greater confidence ratings (mean 4.2, SD 0.6) compared to those with low education (mean 3.8, SD 0.7; *F*_1,74_=8.2; *P*=.01).

### Digital Health Literacy (DHL)

#### Confidence in Tasks

DHL was significantly associated with confidence (*F*_1,74_=5.7; *P*=.02). Participants with high DHL (mean 4.2, SD 0.6) reported higher confidence than those with low DHL (mean 3.9, SD 0.7). Although the Shapiro-Wilk test suggested a minor normality violation (*P*=.04), Q-Q plots showed only slight deviation, the Levene test confirmed homogeneity of variance, and no outliers exceeded the Cook distance of 1.

#### Perceived Difficulty

DHL was significantly associated with perceived difficulty (*F*_1,74_=7.9, *P*=.01). Participants with high DHL reported less difficulty (mean 2.1, SD 0.8) compared to those with low DHL (mean 2.6, SD 0.7).

### Qualitative Data Analysis

FGDs and interviews revealed 5 major themes relevant to the usability and acceptance of the EU Mobile App for Cancer Prevention: accessibility, core features, ethical concerns, motivating and engaging features, and user-friendly design.

#### Accessibility

Participants highlighted the importance of making the app broadly accessible across different cultural, linguistic, and technical contexts. Many emphasized that the app should be available in multiple languages to serve diverse user groups. For example, a participant noted, “It depends on the country... In Cyprus, I think it should have the choice of Greek, English, and Russian because we have many” (Participant 8, Cyprus). Along with multilanguage support, simplicity in language was stressed to accommodate varying literacy levels. As an interviewee stated, “Simple. The simpler the better (Language)” (Participant 6, Cyprus), underscoring the need for plain, nontechnical language. Furthermore, educational content should aim for a 6th-8th grade reading level using plain language guidelines to improve comprehension across literacy levels. In addition, participants were concerned about connectivity issues; offline functionality was deemed critical so that users could continue to access key features even without an internet connection. A participant remarked, “Logging should work even without the Internet” (Participant 1, Slovenia). Together, these insights indicate that ensuring both linguistic inclusivity and offline access are essential for equitable use of the app.

#### Core Features

Participants stressed the necessity of incorporating comprehensive and reliable information about various cancers, including causes, symptoms, and prevention strategies. A participant requested, “Tell me in detail what cancer is in simple words and various symptoms” (Participant 9, Cyprus), reflecting the demand for clear, evidence-based health education. In addition, practical features that aid early detection were highlighted. For instance, an interviewee suggested, “Include screening advice... when you’re consulting screening programs that may be in your community” (Participant 1, Spain), emphasizing the importance of guiding users through regular screenings and self-examinations. Furthermore, resources that support healthier lifestyles, such as tools for tracking physical activity, diet, and other health metrics, were identified as key motivators. A participant observed, *“*Seeing progress happening. that would motivate me to go back there the next day” (Participant 2, Portugal), which illustrates how integrated tracking features can reinforce healthy behaviors.

#### Ethical Concerns

Data protection and privacy emerged as primary ethical issues. Participants highlighted the need for robust security measures to protect personal information and ensure compliance with privacy regulations, such as the General Data Protection Regulation. As a participant explained, “You have to keep all information you get from everyone well-guarded to comply with the European regulation” (Participant 4, Cyprus). This emphasis on data security reflects broader concerns about maintaining user trust. Some participants also expressed that without stringent privacy measures, users might be reluctant to engage with the app, thus impeding its adoption and long-term success.

#### Motivating and Engaging Features

Beyond the provision of core health information, participants emphasized that the app should include features that actively motivate and engage users. A visually appealing, user-friendly design with gamified elements was frequently recommended. A participant noted, “The instructions should be as simple as possible and there should be steps to follow them easily……Very likely with photos, bright colors which are very likely to attract more (users)” (Participant 7, Cyprus), highlighting the role of visual appeal and clear guidance in sustaining user interest. In addition, interactive features that encourage social competition and goal setting were suggested. For instance, a participant commented, “We compete (with friends/family) almost a bit on which one will do several kilometers a month” (Participant 2, Slovenia), demonstrating that competitive and social elements can enhance engagement and adherence to healthy behaviors.

#### User-Friendly Design

Designing the app to be user-friendly across different age groups was seen as essential. For older users, a simplified interface with larger fonts and clear instructions is crucial, while younger users may favor more interactive and gamified elements. A participant stated, “Making it a bit more playful can be beneficial” (Participant 2, Portugal), suggesting that a touch of playfulness could improve usability for younger demographics. In addition, the inclusion of onboarding tutorials was noted as a key feature for easing new users into the app. As another participant remarked, “There are also applications that... with the first use they make you like a small tutorial” (Participant 1, Spain). This reflects the expectation that an intuitive interface with clear navigation, large buttons, and customizable settings will reduce barriers to use and enhance overall accessibility.

## Discussion

### Principal Findings

The study aimed to understand the usability and barriers experienced by different user groups while interacting with a mock version of the EU Mobile App for Cancer Prevention as part of an iterative app development process. Quantitative findings indicated generally high task completion rates and confidence levels; however, significant variations were associated with age, education, and DHL. Qualitative insights further highlighted user preferences and barriers, emphasizing the critical importance of accessibility, user-centered design, ethical considerations, and motivational features. These findings highlight the need for tailored solutions to address the varying needs of diverse user groups.

### Associations of Age, Education, and Digital Health Literacy

User confidence and perceived task difficulty varied by age. Younger adults exhibited higher confidence and reported lower difficulty levels, consistent with literature suggesting greater digital competence among younger individuals [29,30]. In contrast, older adults faced higher difficulty, underscoring the need for age-friendly design elements, such as simplified interfaces, larger fonts, and clear instructions [31]. Importantly, because eHEALS measures self-perceived ability to find, evaluate, and use online health information, lower DHL scores in older participants may reflect reduced confidence rather than objective skill deficits. These findings imply that a one-size-fits-all approach to app design may not be suitable, particularly for older populations who require additional support and resources [32].

Education level and DHL were also linked to user confidence and task difficulty; participants with higher education and DHL scores reported greater confidence and found tasks less challenging. This aligns with studies linking higher digital literacy to better navigation of health tools [30,33]. However, as health literacy data were not collected, we emphasize that educational attainment and DHL are interrelated factors contributing to digital divides [34]. Addressing inequities in DHL (eg, through guided tutorials and simplified navigation) is critical to ensuring equitable access to digital health tools, as these inequities often reflect systemic barriers such as unequal access to technology [35].

### Enhancing Accessibility and Inclusivity

Multilingual support is important to ensure accessibility for non-English speakers and to accommodate the region’s linguistic diversity. Offering the app in multiple languages with high-quality, culturally sensitive translations can significantly enhance comprehension and engagement, as demonstrated in studies of digital health interventions in multicultural settings [29,36]. This approach acknowledges the diverse linguistic backgrounds of European users, making the app more inclusive. Simplified language and clear, jargon-free explanations are essential for users with varying literacy levels, helping to reduce potential barriers to understanding and engagement.

In addition, implementing offline functionality allows users in areas with limited internet access to benefit from the intervention without requiring constant connectivity. This feature is particularly relevant for rural or underserved regions, where reliable internet may not be available [35]. Improving accessibility also involves incorporating features such as adjustable text sizes, voice commands, and compatibility with assistive technologies to accommodate users with disabilities. Incorporating features such as color contrast options, customizable display settings, and alternative text for images can further enhance accessibility for individuals with visual impairments [34]. The goal is to create a flexible and adaptable user interface that caters to the diverse needs of all users, regardless of their physical or cognitive abilities. Addressing these aspects can help reduce the digital divide and promote equitable access to digital health interventions.

### Building Trust Through Ethical Considerations

Robust data protection measures and transparent communication about privacy policies can enhance user trust and willingness to engage with digital health tools. Compliance with regulations like the General Data Protection Regulation (GDPR) is imperative to ensure data security [37]. However, a key dilemma lies in balancing transparency (eg, detailed privacy policies) with user-friendly onboarding processes [38-41]. For instance, lengthy consent forms may deter users, while overly simplified policies risk concealing critical details. Transparency regarding data collection, storage, and usage practices is fundamental to user adoption and engagement, as shown in studies of health app adoption [38-41]. Users need to feel confident that their personal data is being handled responsibly, particularly in the context of sensitive health information. Building trust also involves providing users with control over their data, including options to manage consent and review data-sharing practices. Ensuring that privacy policies are communicated in a clear and understandable manner can help clarify data practices and address user concerns [34].

### Boosting Engagement With Motivational Features

Incorporating motivational and engaging features, such as gamification, social support options, and personalized goal-setting, can promote sustained user engagement and adherence to health-promoting behaviors. Gamification elements, such as rewards, badges, and progress tracking, can make the experience more enjoyable and motivate users to continue using the app, as evidenced in digital health studies [42]. Users particularly appreciated features that actively motivated them, including progress tracking and social interaction elements. Social support features leverage influence and support networks to enhance motivation. Providing options for users to connect with peers, share progress, and receive encouragement can foster a supportive environment that encourages sustained engagement. Simplifying social interaction features and providing guided tutorials or clearer prompts assist users in navigating these aspects effectively, especially those who may not be familiar with social media or online communities [34].

### Providing Educational Content and User-Centric Design

Offering evidence-based, easily understandable information on health-related topics, such as prevention, early detection, and healthy lifestyle practices, is crucial. Educational content must be accurate and sourced from reputable organizations to establish credibility and effectively inform users. In addition to providing general health information, incorporating interactive educational modules (eg, quizzes and videos) can enhance learning and retention, as shown in studies of DHL [42]. Participants specifically requested tutorials and simplified explanations, underscoring the need for iterative, user-centered design. Adopting a culturally sensitive design by involving diverse users throughout the development process allows for feedback and continuous improvements toward enhancing accessibility and relevance. For developers, being mindful of cultural differences in health beliefs and practices enhances the relevance and acceptance of digital health tools across different populations, fostering greater engagement and effectiveness. In addition, iterative pilot testing and refinement based on user feedback are critical to creating a responsive and user-friendly app. Regular updates that incorporate user suggestions can help maintain engagement and ensure that the app continues to meet users’ needs over time. The inclusion of culturally relevant imagery, language, and examples can improve the app’s appeal and effectiveness for diverse user groups.

### Strengths and Limitations of the Study

The sample size was of moderate size for a study using predominantly qualitative methods (n=76) and is likely not representative of the broader European population. However, including participants from 7 European countries and using a mixed methods pilot approach provided valuable preliminary insights. The sampling approach may have introduced selection bias, potentially affecting the generalizability of the findings. The use of mock wireframes instead of a fully functional app could not fully capture user interactions and potential usability issues present in the final app. To enhance methodological rigor, we used the validated eHEALS instrument to assess DHL. We acknowledge that the eHEALS captures self-rated confidence in locating, appraising, and applying online health information rather than objectively evaluating digital health skills. Although it is the most widely validated instrument for perceived electronic health literacy, its reliance on subjective self-assessments may not fully correspond to actual proficiency in using digital health resources. In addition, the cross-sectional design limits the ability to assess long-term engagement and behavior change resulting from app use. Future studies should consider larger, more diverse samples to improve generalizability. Using a longitudinal design could also provide insights into how user engagement and behavior change evolve over time. Furthermore, incorporating a fully functional app in future usability testing will allow more accurate assessment of real-world user interactions and barriers.

### Future Directions

This initial usability study provides a foundation for further development and refinement of the app. Following this pilot, we delivered a comprehensive summary of identified enablers and barriers to the app development team and conducted multiple collaborative iterative meetings. As a result, the next prototype incorporated larger and more intuitive navigation elements, additional language support, an interactive onboarding tutorial, and a settings screen, ensuring that user feedback directly shaped the app’s evolution before its live testing in the next phase. The next phase will involve piloting the developed app by distributing it to participants for installation on their personal devices [43]. This real-world testing will allow users to interact with the app and provide feedback on functionality, engagement, and impact on health behaviors before making it available to EU citizens [43]. Future research should focus on strategies to enhance DHL among users, further improving engagement and effectiveness. This could include incorporating educational modules that improve users’ digital competence and offering community support programs to assist users in effectively using digital health tools [34].

Integration with health care systems presents another promising direction. Collaborating with health care providers to align digital health tools with existing health services could enhance their usability and impact [44]. Including features that allow health care professionals to provide input or feedback may increase the credibility and effectiveness of digital interventions. Features such as secure messaging, cancer screening appointment scheduling, and health data sharing could foster greater integration with traditional health care services. Engaging with policymakers to promote supportive regulations and standardization efforts can facilitate broader adoption and integration of digital health tools. Policymaker engagement is also critical to addressing systemic barriers to digital health adoption, such as inadequate infrastructure and lack of digital literacy programs [34].

### Conclusion

The pilot usability testing of the EU Mobile App for Cancer Prevention indicates that while the app is generally user-friendly, optimizing its effectiveness across diverse populations requires careful consideration of age, educational attainment, and DHL. Incorporating user feedback—particularly on accessibility features, ethical considerations, and engaging content—is crucial for enhancing adoption and promoting equitable access to cancer prevention resources. By focusing on social factors and equity, developers can create a cancer prevention digital health intervention that is more effective, inclusive, and responsive to the diverse needs of the population. Addressing the barriers identified in this study can significantly strengthen the app’s role in supporting Europe’s Beating Cancer Plan and reducing the cancer burden across the European Union.

## Supplementary material

10.2196/73844 Multimedia Appendix 1 Recruitment strategy and interview guide.

## Abbreviations

AI: artificial intelligence
BUMPER: Boosting the Usability of the EU Mobile App for Cancer Prevention
DHL: digital health literacy
ECAC: European Code Against Cancer
eHEALS: eHealth Literacy Scale
FGD: focus group discussion
