# Supplementary material for: Assessing the User Experience of the EU Mobile App for Cancer Prevention: Mixed Methods Study
Source: JMIR Form Res. 2025 Sep 19;9:e73844. doi: 10.2196/73844 (PMC12448259; doi:10.2196/73844)
Supplement: Multimedia Appendix 1 [file formative-v9-e73844-s001.docx]

**Recruitment Strategy**

Participants were recruited using a purposive sampling strategy to ensure a diverse cohort across age groups, gender, and education levels in each country. Efforts were made to adhere closely to this strategy, aiming for an equal distribution of participants across the specified demographic categories.

| **Age Group** | **Gender** | **Education Level*** | **Number of Participants** |
| --- | --- | --- | --- |
| 18-40 | Men | low- high | 2 |
| 18-40 | Women | low- high | 2 |
| 41-60 | Men | low- high | 2 |
| 41-60 | Women | low- high | 2 |
| 61 and above | Men | low- high | 2 |
| 61 and above | Women | low- high | 2 |

** low: no formal education, primary, lower or upper secondary; high: trade/technical/vocational, bachelor’s degree, master’s degree, doctorate*

Interview guide

- The interviewer will give an overview of the project and the research team.
- The interviewer will explain the project's broad goals as well as the scope of the study and why it is being undertaken.
- The interviewer will take informed consent, if not taken earlier, and then will explain and remind the participant that they can decline to answer any questions and remove consent at any time during the interview.
- After the entry question the interviewer will present some screenshots of the app prototype.

| Topic | Probe Questions (Essential) | Follow-up Questions (Used to explore the topic) |
| --- | --- | --- |
| Entry Questions | What would you expect from an App that promotes cancer prevention? | Which topics are the most important ones? |
| User friendliness and usability | How can we make the app easy to use so that people are more likely to use it? | What specific features or design elements do you think would make the app more user-friendly?  Have you used any apps that you found particularly easy or difficult to use, and can you tell me why? |
| Maintenance | What are features that will motivate people to use the app over a longer period of time? | Can you think of an app that you have user regularly over a longer period of time? What was special about this app in contrast to other apps? |
| Languages | What level of language should be used in terms of how easy it is to read? | Are there any specific languages or language groups that the app should be made available in? Why? |
| Internet Connectivity | Do you think there would be any problems if the app cannot be used without internet connectivity? | Are there any features of the app that you think should still be accessible without an internet connection? Why? |
| Capacity and resources | Can you think of any capabilities, expertise, and resources that users might need to use the app? | Can you think of any equipment, supplies, training resources, or information about how to use the app that needs to be given to the target audience or the general public? |
| Data security and protection | When we talk about data security and data protection, what do you think we should think about when developing the app? | Can you give an example of a data security or protection concern that should be addressed in the app? How do you think this concern might impact the end users or you? |
| Ethical issues | Can you think of any ethical issues that might be a concern to the end users including you of the cancer prevention app that we should consider? | How do you think this issue might impact the end users/you? |
| Inter-operability | How do you think this app should be made available to users? | Are there any specific platforms or devices that you think the app should be available on? |

**EQUATOR Checklist (STARE-HI)**

| **STARE-HI item** | **Item comprised in manuscript? (√/–)** | |
| --- | --- | --- |
| **3.1 Title** | Clear, descriptive title | √ |
| **3.2 Abstract** | Structured abstract covering background, methods, results, conclusions | √ |
| **3.3 Keywords** | Appropriate indexing keywords | √ |
| **3.4 Introduction** |  | √ |
| **3.4.1 Scientific background** | Context and literature review | √ |
| **3.4.2 Rationale for the study** | Gap identification and justification | √ |
| **3.4.3 Objectives of the study** | Explicit study aims | √ |
| **3.5 Study context** |  | √ |
| **3.5.1 Organizational setting** | Description of sites (Living Lab, Cancer Leagues) | √ |
| **3.5.2 System details and system in use** | Mock wireframe prototype description, tabs, navigation | √ |
| **3.6 Methods** |  | √ |
| **3.6.1 Study design** | Mixed-methods pilot design | √ |
| **3.6.2 Theoretical background** | Underlying digital public health framework | √ |
| **3.6.3 Participants** | Eligibility, sampling, recruitment procedures | √ |
| **3.6.4 Study flow** | Training, wireframe tasks, FGD sequence | √ |
| **3.6.5 Outcome measures or evaluation criteria** | Usability metrics, eHEALS, confidence/difficulty ratings | √ |
| **3.6.6 Methods for data acquisition and measurement** | Task timing, Likert scales, audio recording | √ |
| **3.6.7 Methods for data analysis** | Quantitative (ANOVA) and qualitative (thematic) analysis | √ |
| **3.7 Results** |  | √ |
| **3.7.1 Demographic and other study coverage data** | Participant demographics table | √ |
| **3.7.2 Unexpected events during the study** | — | – |
| **3.7.3 Study findings and outcome data** | Task performance, DHL scores, themes | √ |
| **3.7.4 Unexpected observations** | — | – |
| **3.8 Discussion** |  | √ |
| **3.8.1 Answers to study questions** | Interpretation of usability enablers/barriers | √ |
| **3.8.2 Strengths and weaknesses of the study** | Mixed-methods strengths, limitations (eHEALS, wireframes) | √ |
| **3.8.3 Results in relation to other studies** | Comparison with literature on age, DHL, app design | √ |
| **3.8.4 Meaning and generalizability of the study** | Implications for EU app rollout, equity considerations | √ |
| **3.8.5 Unanswered and new questions** | Future directions, need for objective measures | √ |
| **3.9 Conclusion** | Broader conclusions and recommendations | √ |
| **3.10 Authors’ contribution** | Contributor roles table | √ |
| **3.11 Competing interests** | Declaration of no conflicts | √ |
| **3.12 Acknowledgement** | Funding and project acknowledgments | √ |
| **3.13 References** | Complete, formatted reference list | √ |
| **3.14 Appendices** | Supplementary File 1 (interview guide), checklists | √ |

*(The above checklist follows the STARE-HI guidelines for reporting health informatics evaluations, adapted to this mixed-methods usability study.)*
